# Novel tests of capture by irrelevant abrupt onsets: No evidence for a mediating role of search task difficulty during color search

**DOI:** 10.3758/s13414-022-02623-y

**Published:** 2022-12-02

**Authors:** Rebecca Rosa Schmid, Ulrich Ansorge

**Affiliations:** 1 Department of Cognition, Emotion, and Methods in Psychology, Faculty of Psychology, University of Vienna, Liebiggasse 5, 1010 Vienna, Austria; 2 Vienna Cognitive Science Research Hub, University of Vienna, Vienna, Austria; 3 Research Platform Mediatised Lifeworlds, University of Vienna, Vienna, Austria

**Keywords:** Color search, Attentional capture, Attentional dwelling, Salience, Event-related potentials

## Abstract

According to the attentional dwelling hypothesis, task-irrelevant abrupt-onset cues capture attention in a stimulus-driven way by eliciting spatial shifts and further dwelling at cue position until target onset. Consequently, search can be facilitated for targets at cued locations relative to uncued locations. Critically, effects of stimulus-driven capture can go undetected in mean reaction times and error rates when search is too easy. In contrast, according to the priority accumulation framework (PAF), cueing effects for task-irrelevant cues differ from cueing effects by task-relevant cues. Most critically, cueing effects by irrelevant cues do not necessarily index spatial shifts and more dwelling but rather retrieval of cueing information. We used both behavioral measures (i.e., cueing effects and distractor compatibility effects) and event-related potentials on direct visual orienting activity elicited by the cue (Experiment 2) as well as consequences on target processing (Experiment 1) to investigate whether task-irrelevant abrupt onsets elicited attention shifts and led to further dwelling. We found behavioral support for attentional effects of task-irrelevant cues, surprisingly, however, only when search displays remained on-screen until response. We found no support for the attentional dwelling hypothesis or for PAF in the size of cueing effects as a function of search difficulty. Critically, lateralized ERPs revealed that salience of abrupt onsets per se is not sufficient to elicit spatial shifts during color search. Finally, neurophysiological evidence demonstrates that choices toward the implementation of experimental protocols can dramatically alter behavioral results on attentional effects of salient, but task-irrelevant abrupt onsets and conclusions drawn from them.

**Supplementary Information:**

The online version contains supplementary material available at 10.3758/s13414-022-02623-y.

## Introduction

Humans can exert top-down control over their selection of visual information to focus on information relevant to their current goals (e.g., Folk et al., 1992). However, human attention might still be captured in a stimulus-driven way by salient but task-irrelevant events, such as abrupt onsets (e.g., Folk & Remington, 2015; for a recent review, see Luck et al., 2021). Take the example of cueing studies. During visual search for color-defined targets, presenting an irrelevant abrupt-onset cue prior to a target, many studies found no cueing effects: Response times (RTs) were the same, regardless of whether the cue was presented at target location (in the valid condition) or away from it (in the invalid condition; e.g., Folk et al., 1992; Goller et al., 2020). This was interpreted as evidence against stimulus-driven capture of attention, as otherwise one would have expected a cueing (or validity) effect—facilitated target search in valid compared with invalid conditions. Critically, under relatively similar conditions, top-down matching cues of the searched-for color showed significant cueing effects (e.g., Anderson & Folk, 2010; Folk et al., 1992; Goller et al., 2020).

Maybe, however, search for a color target is sometimes too simple. This was suggested by the attentional dwelling hypothesis (Gaspelin et al., 2016). Gaspelin et al. (2016) proposed that abrupt-onset cues would always capture attention in a stimulus-driven way by eliciting covert spatial shifts toward those cues. Attention would then dwell at the cued location until target-display onset. However, when the target can be easily found due to a large color difference between target and distractors, humans would swiftly disengage attention from an invalidly cued distractor and allocate their attention to the targets, essentially masking any behavioral cueing effect. Only during difficult search, attention would continue to dwell at the distractor location following target display onset, eventually giving rise to a behavioral cueing effect (cf. Bacon & Egeth, 1994; Folk et al., 1992; Goller et al., 2020; Lien et al., 2008; Theeuwes, 2010; Yantis & Jonides, 1990).

The prediction on covert spatial shifts in the cue-to-target time interval can be tested best by means of recording neurophysiological data, specifically, event-related potentials (ERPs) of the electroencephalogram (EEG). Admittedly, some past studies found that cues only elicited reliable ERP markers of attentional capture (as well as a behavioral cueing effect) if the cues matched the searched-for target color and, thus, carried a task-relevant feature (e.g., Arnott et al., 2001; Goller et al., 2020; Lien et al., 2008). For example, Goller et al. (2020) found a cue-elicited N2-posterior-contralateral component (N2pc)—a lateralized ERP component with an onset of about 200 ms post-cue that is more negative contralateral than ipsilateral to the cue (cf. Luck & Hillyard, 1994)—only following target-similar and, hence, relevant or top-down matching abrupt-onset cues (e.g., of a red abrupt-onset cue during search for red targets). In contrast, task-irrelevant abrupt-onset cues of a color different than the target color (e.g., green abrupt-onset cues during search for red targets) did neither produce an attention-related N2pc effect nor a behavioral cueing effect (Goller et al., 2020).

However, past ERP studies of capture by abrupt onsets only used easy color search (e.g., Arnott et al., 2001; Goller et al., 2020). This is a noteworthy gap in the literature, although, according to the attentional dwelling hypothesis, a task-irrelevant abrupt-onset cue should have triggered attention orienting activity under easy color search conditions, too. To explain this discrepancy, at variance with the attentional dwelling hypothesis but in line with a moderating role of task difficulty, an attention-related ERP effect and/or behavioral cueing effect might simply take more time to build up than is typically available under easy search conditions, and more evidence for stimulus-driven attention capture could, thus, be observed under more difficult search conditions (cf. Gaspelin et al., 2016). Therefore, the current study also used a difficult color search condition to see if more evidence for stimulus-driven attention capture by irrelevant abrupt-onset cues can then be found in attention-related ERP markers and cueing effects.

Here, we set out to test four predictions related to the attentional dwelling hypothesis. First, in Experiment 1, we originally intended to study cue-elicited lateralized ERPs to see if the irrelevant onset cues elicited such lateralized attention-related ERPs (i.e., an N2pc) at least under difficult search conditions. This rationale was based on Goller et al. (2020), who observed no sensory lateralization and only an N2pc in response to relevant abrupt-onset cues of the searched-for target color. However, in contrast to Goller et al. (2020), in the current Experiment 1, cues already elicited several lateralized ERPs prior to the N2pc time window. As we were not able to decide if these lateralized ERPs reflected sensory imbalances or attentional effects of the cues, we eventually abstained from analyzing cue-elicited ERPs in Experiment 1, but the goal was picked up more generally again in Experiment 2 (see below).

Second, however, in Experiment 1, we investigated cue-relative ERP modulations of target processing to draw inferences concerning attentional effects of target-preceding cues. Past studies have shown that sensory-evoked ERPs in response to a stimulus at an attended-to location were amplified (e.g., Eimer, 1994; Hillyard & Anllo-Vento, 1998; Hopfinger & Mangun, 1998; Hopfinger & Ries, 2005; Mangun, 1995; Mangun & Hillyard, 1991). This effect of attention (in the present study, corresponding to a cueing effect and, therefore, called the *ERP cue validity effect*) is measured as an increased amplitude in the lateralized occipital components (i.e., P1/N1 peaks) for targets at attended (here, validly cued) locations. This ERP marker is considered to reflect *sensory gain control* (e.g., Hillyard et al., 1998; Mangun & Hillyard, 1995), and it is assumed to correspond to improved perceptual acuity at the attended location (e.g., Eimer, 1994; Luck et al., 2000; Mangun, 1995). Hence, following the attentional dwelling explanation, stating that salient, but task-irrelevant, peripheral cues elicit spatial shifts in a stimulus-driven way and attention further dwells at the cued location until search display onset, we expected to find evidence for sensory gain control for targets at validly cued locations, but not for targets at invalidly cued locations, reflected in an ERP cue validity effect.

Third, in Experiment 2, we investigated attention-related ERP markers of direct visual orienting activity elicited by the cue rather than the cue’s mere consequences on target processing. To note in Experiment 1, the sudden onset of one unilateral cue caused ERPs possibly reflecting lateralized sensory rather than attentional influences (Luck, 2012). To decide if some of the lateralized ERPs reflected attentional effects, in Experiment 2, we included task-relevant/top-down matching cueing conditions (with abrupt-onset cues of the searched-for color) in addition to irrelevant abrupt cues, but with both cue types being of exactly the same energy (luminance or contrast). Task-relevant/top-down matching cues are known to reliably elicit attention capture effects (e.g., Goller et al., 2020; Lien et al., 2008). Thus, if some of the cue-elicited lateralized ERPs reflected visual attention, we expected differences between the task-relevant and the task-irrelevant cue-elicited lateralized ERPs. If abrupt onset cues elicited attention shifts under difficult but not (or less so) under easy color search conditions, we should be able to see more such attention-related lateralized ERP components in an ERP difference between task-relevant and easy/task-irrelevant conditions than in a difference between task-relevant and difficult/task-irrelevant conditions. Of course, it is only assumed that task-relevant cues elicit more attention capture and lateralized ERPs. It is, however, also possible that all cues—relevant and irrelevant abrupt onset cues—are equally effective in capturing spatial attention, in which case no differences might be observed and, we could not decide if attention contributed to the cue-elicited ERPs in any of the conditions. In addition, based on the attentional dwelling hypothesis, we expected no differences between the lateralized ERPs under easy/task-irrelevant and under difficult/task-irrelevant conditions, meaning that we could not use the difference between the cue-elicited ERPs in these two conditions of Experiment 2 to confirm the attentional dwelling hypothesis.

Lastly, we evaluated another prediction of the attentional dwelling hypothesis: that attention was engaged at a cued location (cf. Carmel & Lamy, 2014; Folk & Remington, 2006; Theeuwes et al., 2000). To that end, we analyzed distractor-compatibility effects (next to cueing effects): We tested if the response feature presented at an invalidly cued distractor influenced the target reaction time (RT). To note, distractors either shared or did not share a response-relevant feature with the targets, so that invalidly cueing a distractor with a feature response-compatible to the target could speed up target RT relative to a distractor carrying a response-incompatible feature. If attention indeed dwells at the cued location and continues to do so into the target displays under difficult search conditions as the attentional dwelling hypothesis claims, then one would expect a response-compatibility effect based on distractor information available at the invalidly cued distractors. For example, Zivony and Lamy (2018) ran a variant of Gaspelin et al.’s (2016) difficult color search protocol similar to the one we used in the current study, with abrupt-onset cues that either shared the target color (task-relevant, top-down matching) or did not share the target color (task-irrelevant, stimulus-driven capture). In contrast to Goller et al. (2020), they found significant cueing effects for both task-irrelevant and task-relevant cues. In line with the long-standing interpretation of cueing effects as covert attention shifts, this can be interpreted as an indication that both cues affected spatial orienting (but see the Discussion for alternative interpretations of cueing effects in terms of influences on posttarget processing rather than on attention capture). Interestingly, however, Zivony and Lamy found that only task-relevant cues elicited significant distractor-compatibility effects (i.e., faster RTs in invalid trials in which the cued distractor’s response-related feature and the target’s response-related feature were the same) indicating a deeper processing and attentional engagement at the cued distractor’s location with task-relevant than task-irrelevant cues (but see Büsel et al., 2021, for such compatibility effects even with task-irrelevant abrupt-onset cues). Zivony and Lamy concluded that involuntary capture by task-irrelevant cues might not have recruited all forms of spatial attention and, thus, that at least attentional engagement is contingent on human search goals. In light of discrepant findings (Büsel et al., 2021 vs. Zivony & Lamy, 2018), we here put this conclusion to another test.

## Experiment 1

### Materials and methods

#### Participants

Twenty-three undergraduate psychology students from the University of Vienna took part, either in exchange for course credits or monetary compensation (18 females; *M*_age_ = 20.0 years, *SD*_age_ = 2.9 years, range: 19–29 years). The sample size was based on an a priori power calculation with G*Power (Version 3.1.9.4) assuming a medium effect size (*f* = 0.25) and a statistical power of 80% and an alpha level of 5%. Participants were right-handed, had normal or corrected-to-normal visual acuity, and normal color vision. They were naïve to the purpose of the experiment and treated in accordance with the standards of the Declaration of Helsinki. We further followed the Austrian Universities Act, 2002 (UG2002, Article 30 § 1), which states that only medical universities or researchers conducting applied medical research are required to obtain an additional approval by an ethics committee. As our study also did not use deceit, no additional ethical approval was required for our study. We determined a priori that only participants with a maximum of 20% error rates would be included in the analyses. Based on this criterion, no datasets were excluded. We further determined a-priori that only neurophysiological data from participants with less than 20% of trial rejections due to artifact contamination and less than 10% of channel interpolation due to noisy signal would be included in the ERP analysis. Further, the interpolation of one of the channels of interest (i.e., PO7 or PO8) led to exclusion of the respective dataset. Based on these criteria, five datasets were excluded, leaving neurophysiological data from 18 participants for the ERP analysis (13 females; *M*_age_ = 20.8 years, *SD*_age_ = 2.5 years, range: 19–28 years).

#### Experimental setup and task

Visual stimuli were presented on a 19-in. CRT monitor (Sony Multiscan) with an aspect ratio of 4:3, a resolution of 1,280 × 1,024 pixels, and a refresh rate of 100 Hz. Participants sat comfortably in front of the monitor in a soundproof, dimly lit room. The distance between their eyes and the screen was kept constant at 57 cm by a chin rest. The experiment was presented via OpenSesame (Version 3.2.8; Mathôt et al., 2012) on a PC running Windows 7.

**Fig. 1:**
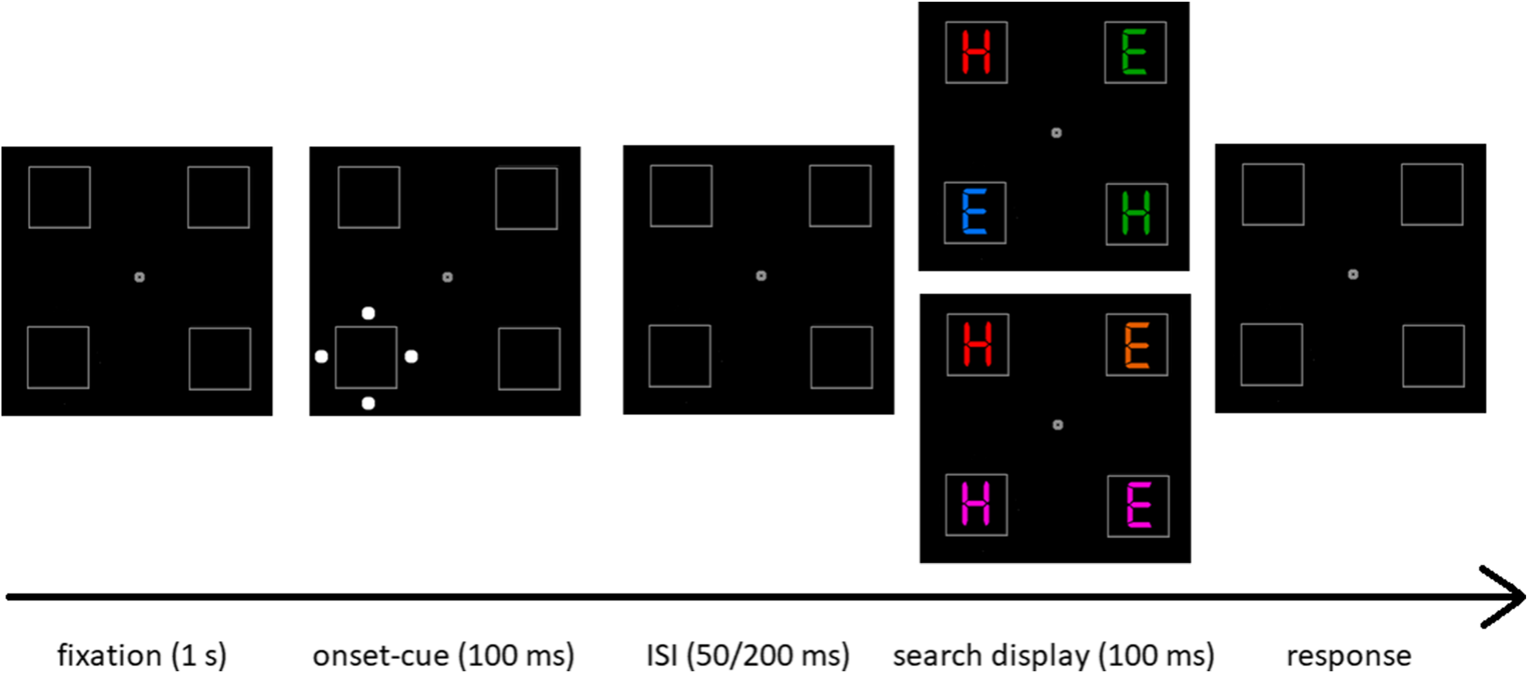
Experimental setup for Experiment 1. *Note.* Following a fixation screen, a white onset cue was presented at one of the four placeholders. Following an interstimulus interval (ISI), a search display was presented. Participants were required to respond to red target letters by key press - *m* key for red *H*s and *y* key for red *E*s, respectively. Top (2^nd^ box from right): Exemplary trial of the easy color search condition (i.e., distractor colors were blue and green). Bottom (2^nd^ box from right): Exemplary trial of the difficult color search condition (i.e., distractor colors were pink and orange). Note that stimuli are not drawn to scale and in brighter colors than in the original experiment. (Color figure online)

Figure 1 illustrates the experimental setup. All stimuli were presented centered on the screen against a black background (CIE L*a*b 1.4/1.3/−1.5). Four unfilled rectangular 2.4° (width) × 2.4° (height) boxes—serving as placeholders at the corners of an imaginary 10° (width) × 10° (height) square, as well as a small fixation dot at center with 0.18° (diameter) were displayed throughout the experiment (all grey; CIE L*a*b 52.2/−3.7/−24.1). Each trial started with a 1-s fixation period. In the cueing display, four white dots (CIE L*a*b 97.2/−3.6/−34.9; 0.5° in diameter) abruptly onsetted for 100 ms around one of the four placeholders forming an imaginary 3.3° (width) × 3.3° (height) diamond. Which of the four placeholders changed per each of the trials varied pseudo-randomly. After an interstimulus interval (ISI) of either 50 ms or 200 ms, a target(-search) display was presented. Interval was varied, as (1) a short cue-target ISI corresponds best with cue-target ISIs used in past studies but (2) a longer cue-target ISI allows measurement of the cue-elicited ERPs free from target-elicited ERPs, and (3) a comparison of cue-elicited ERPs under short and long ISIs may, thus, help to tell cue-elicited from target-elicited ERPs if this were necessary. Following the ISI, the target(-search) display consisted of three colored distractor letters and one colored target letter (always red) presented for 100 ms. Letters were either an *H* or an *E* in a digital number font at 1.9° (width) × 1.9° (height) in equiluminant colors. To manipulate the search difficulty, between conditions, we varied the distance of the distractors in color space from the red target (CIE L*a*b 51.2/69.0/59.7). In the easy color search block, the red target was presented far away in color space from the green (CIE L*a*b 51.1/−58.4/48.4) and blue (CIE L*a*b 51.0/25.1/−99.4) distractors. In the difficult color search block, the red target was presented nearer in color space to the pink (CIE L*a*b 51.1/69.0/−8.8) and orange distractors (CIE L*a*b 51.2/41.7/57.0). Per each trial, two distractor colors were randomly assigned to the three distractors, with the restriction that both distractor colors were far away or that both were nearer to the target (e.g., two green and one blue), so as to prevent a top-down singleton-search mode (cf. Bacon & Egeth, 1994). To prevent participants using shape instead of color to find the target, both target letter identities were randomly assigned to the stimulus positions, with the restriction that each display contained two *E*s and two *H*s. These shapes were the response-relevant features of the targets (see below).

The participants’ task was to search for the red target letter throughout the experiment and respond as fast and as accurately as possible by pressing the *m* key for red *H*s and *y* key for red *E*s, by using their right versus left index fingers, respectively. Participants were informed that the cue’s position was not predictive for the position of the target and could, thus, be safely ignored. Responses could be given within 2 s following target offset. Participants received visual feedback, if responses were incorrect (“Falsch”; i.e., “wrong” in German) or slower than 1.5 s (“Schneller antworten”; i.e., “respond faster” in German), and these trials counted as errors. Additionally, participants received visual feedback on their average RTs (ms) and correct answers (%) during all self-paced breaks after every 100 trials. Prior to the experimental blocks, each participant practiced the task for both search difficulties (32 trials for easy color search and 32 for difficult color search). If either an incorrect response or a response slower than 1.5 s was given, respective trials were repeated at the end of the practice block. The experiment consisted of one easy color search block and one difficult color search block. Additionally, we added one difficult color search block with a slightly longer ISI (i.e., 200 ms instead of 50 ms). Each block consisted of 400 trials, thus, resulting in 1,200 trials in total. The block order was randomized between and balanced across participants.

#### Electroencephalographic data recording and processing

EEG data was recorded at 512 Hz with an ActiveTwo Biosemi™ electrode system (BioSemi B.V., Amsterdam, Netherlands) from 132 electrodes (128-scalp electrodes) mounted in an equiradial ABC layout. The ground electrode at AFZ served as an online reference. The vertical and horizontal electro-oculogram was recorded on the right eye from approximately 3 cm below the eye and from the outer canthi, respectively. An additional two electrodes were placed bilaterally on the mastoids. Electrode impedances were always kept below 20 kΩ. Note that the used system tolerates high impedances and is, therefore, capable to supply high data quality beyond the usual recommendations on impedance maxima (cf. Kappenman & Luck, 2010). EEG data were processed in MATLAB (v9.6; The MathWorks Inc., 2019) using the EEGLAB toolbox (Delorme & Makeig, 2004) with the ERPLAB extensions (Lopez-Calderon & Luck, 2014). In agreement with conventions, the signal was resampled offline to 128 Hz, rereferenced to the average of both mastoid channels, high-pass filtered with a finite impulse response filter (FIR) at 0.1 Hz with a cutoff frequency of 0.05 Hz (−6 dB) and a transition band width of 0.1 Hz. After epoching, any contaminated channels were interpolated. Next, the epoched EEG data was subjected to a commonly used artifact rejection procedure for a time interval from −100 ms (prior to stimulus onset) to 400 ms (following stimulus onset). The corresponding epoched data was rejected when the EEG exhibited high signal changes (more than 50 μV/ms), low activity (less than 0.5 μV difference between subsequent samples for a time period of at least 500 ms), if overall values exceeded ± 100 μV in any channel, or if the EOG exceeded ± 80 μV. To assess residual eye movements, we computed averaged HEOG waveforms for left and right stimulus trials. We excluded any dataset in which the residual EOG activity was more than 3.2 μV, which means that the residual eye movements in the remaining participants were less than 0.2° with propagated voltage of less than 0.1 μV at the posterior scalp sites (i.e., PO7 and PO8; Lins et al., 1993). Among the final set of 18 participants, 11.7% of all trials were removed by this procedure. Baseline correction was performed for the entire epoch with respect to the 200 ms interval before cue onset. For illustrations, ERP data was low-pass filtered at 30 Hz.

### Results

Data was statistically analyzed in R 4.1.0 (R Core Team, 2021) using R studio (v1.3.1093; RStudio Team, 2020) and the ez package (v4.4-0; Lawrence, 2016). Trials with RTs less than 100 ms or greater than 2.5 standard deviations of the participant’s mean RT (2.2% of all trials) were excluded from all analyses (cf. Goller et al., 2020). Trials with erroneous answers were excluded from the analyses of RTs and ERPs. Besides *p* values, we additionally reported Bayes factors (BF; Rouder et al., 2009) for all critical pairwise comparisons to provide information about the relation between the probabilities of the data being in favor of the null relative to being in favor of the alternative hypothesis. On the basis of Jeffreys' (1961) convention, a BF greater than 3.00 is considered substantial evidence in favor of the alternative hypothesis (Dienes, 2014).

#### Attentional capture (cueing effects)

For the analysis on attentional capture by the cues (Gaspelin et al., 2016), a repeated-measures analysis of variance (ANOVA), with the within-subject variables *search condition* (easy color search with short ISI vs. difficult color search with short ISI vs. difficult color search with long ISI) and *validity* (invalid cue vs. valid cue) was conducted on mean correct RTs and arcsine-transformed error rates, respectively.

##### Response times

The ANOVA showed a significant main effect of *search condition* only, *F*(2, 44) = 5.16, *p* = .026, η_p_^2^ = .19. Participants responded more quickly during easy color search (505 ms) than difficult color search (531 ms with short ISI; 524 ms with long ISI), but with a Bonferroni correction for three comparisons (α = .017), only the observed differences between conditions easy color search and difficult color search with long ISI were significant, *t*(22) = −2.90, *p* = .008, *d* = 0.347, otherwise, all *t*s < 2.30, all *p*s > .031, all *d*s < 0.45.

The key question in regard to Gaspelin et al. (2016) was whether the size of cueing effects was dependent on search difficulty (see Table 1 for all mean RTs by *search condition* and *validity*), but the ANOVA did neither yield a significant interaction of *search condition* and *validity*, *F*(2, 44) = 0.63, *p* = .539, η_p_^2^ = .03, nor a significant main effect of *validity, F*(1, 22) = 2.79, *p* = .109, η_p_^2^ = .11, at all.^1^ This result was further supported by a planned comparison showing no systematic differences in the size of cueing effects between easy and difficult color search conditions, *t*(22) = 0.91, *p* = .371, *d* = 0.03, *BF*_10_ = 0.22.

**Table 1:** Mean response times (ms) by search condition and validity

	Easy color search (short ISI)	Difficult color search (short ISI)	Difficult color search (long ISI)
Invalid	506	532	525
Valid	500	526	522
Cueing effect	6	6	3

Cueing effects were calculated as invalid minus valid performance

##### Error rates

Participants made numerically more errors in difficult color search (5.1% with short ISI; 4.5% with long ISI) than easy color search (3.8%), but observed differences were not significant, *F*(2, 44) = 2.29, *p* = .113, η_p_^2^ = .09. As in mean RTs, the interaction of interest between *search condition* and *validity* was also not significant, *F*(2, 44) = 0.12, *p* = .884, η_p_^2^ = .01.

#### Attentional engagement (distractor compatibility effects)

For the analysis of attentional engagement reflected in distractor-compatibility effects (Zivony & Lamy, 2018), a repeated-measures ANOVA, with the within-subject variables *search condition* (easy color search with short ISI vs. difficult color search with short ISI vs. difficult color search with long ISI) and *distractor compatibility* (compatible response vs. incompatible target-response with cued distractor) was conducted on mean RTs and arcsine-transformed error rates. Trials in which the target appeared at the same location as the preceding cue (i.e., a valid cue) were excluded from this analysis.

##### Response times

The ANOVA showed a significant main effect of *search condition* only, *F*(2, 44) = 5.14, *p* = .026, ηp^2^ = .19. Participants responded generally faster in easy color search (506 ms) than difficult color search (532 ms with short ISI; 526 ms with long ISI), but post hoc *t* tests (α = .017; Bonferroni corrected for three comparisons) showed only significant differences between conditions of easy color search and difficult color search with a long ISI, *t*(22) = −2.79, *p* = .0107, *d* = 0.326, otherwise all *t*s < 2.32, all *p*s > .030, all *d*s < 0.45.

The key question in regard to Zivony and Lamy (2018) was whether distractors which appeared at the cued location in the target display generated a distractor-compatibility effect. Here, the ANOVA did neither yield a significant main effect of *distractor compatibility, F*(1, 22) = 0.29, *p* = .597, η_p_^2^ = .01, nor a significant interaction of *search condition* and *distractor compatibility*, *F*(2, 44) = 0.98, *p* = .383, η_p_^2^ = .04 (see Table 2 for all mean RTs by *search condition* and *distractor compatibility*).

**Table 2:** Mean response times (ms) by search condition and distractor compatibility

	Easy color search (short ISI)	Difficult color search (short ISI)	Difficult color search (long ISI)
Incompatible	507	532	526
Compatible	506	533	522
Compatibility effect	1	−1	3

Compatibility effects were calculated as incompatible minus compatible performance

##### Error rates

Participants made numerically more errors in difficult color search (5.2% with short ISI; 4.5% with long ISI) than easy color search (3.8%), but the observed differences were not significant, *F*(2, 44) = 3.31, *p* = .059, η_p_^2^ = .13. Again, the ANOVA did neither yield a significant main effect of *distractor compatibility, F*(1, 22) = 0.29, *p* = .597, η_p_^2^ = .01, nor a significant interaction between both factors, *F*(2, 44) = 0.57, *p* = .571, η_p_^2^ = .03.

#### Event-related potentials

**Fig. 2:**
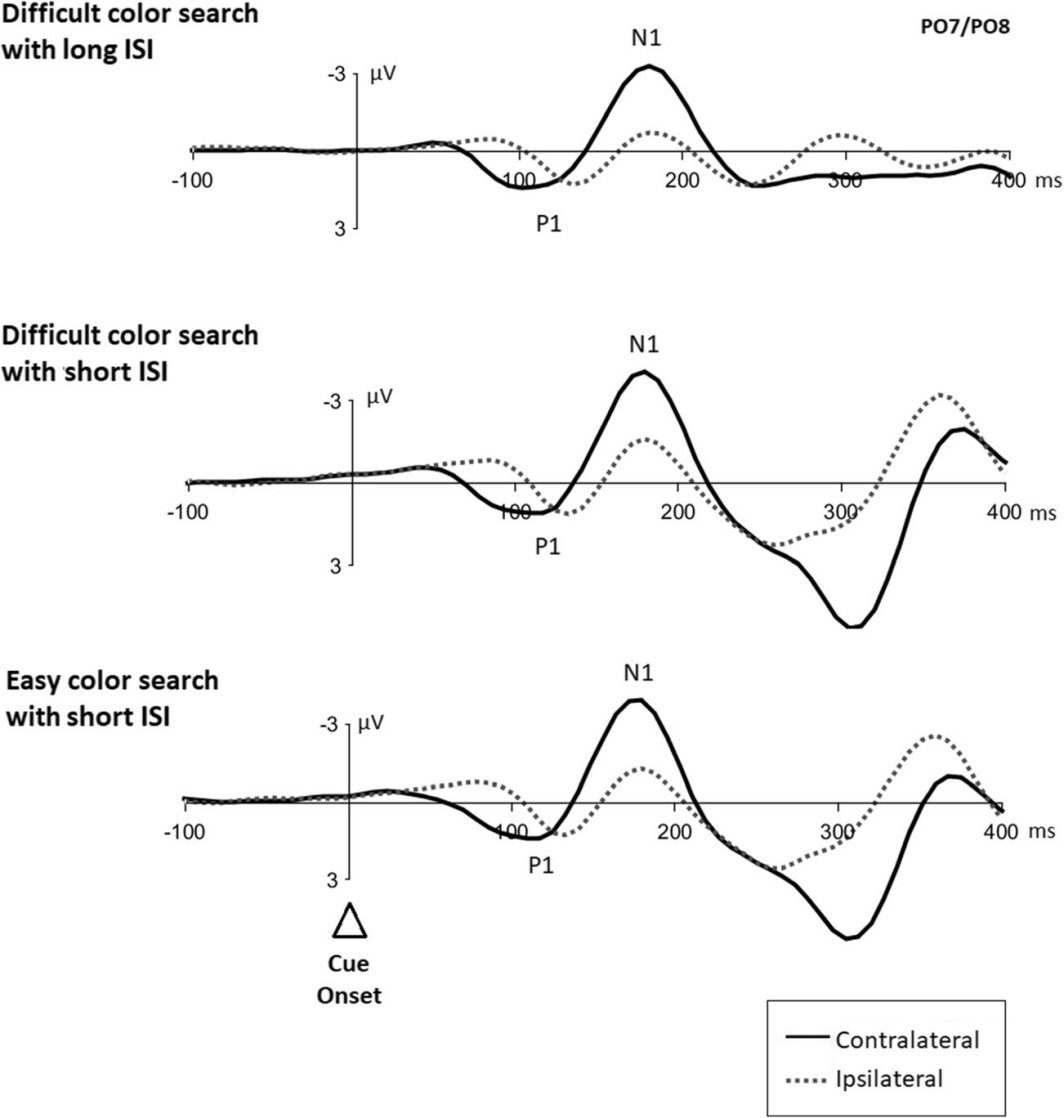
Cue-elicited event-related potentials from each search condition. *Note.* Event-related potentials (ERPs) in difficult color search with long interstimulus intervals (ISI, top), difficult color search with short ISI (middle), and easy color search with short ISI (bottom) plotted time locked to cue onset. All graphs show the ERPs at posterior electrode sites PO7/PO8 with a respective baseline of 200 ms prior to cue onset. Negative values are plotted upward. The dotted lines illustrate the ipsilateral ERPs, the solid lines the contralateral ERPs

Figure 2 shows cue-elicited visual ERP waveforms at posterior contralateral and ipsilateral electrode sites (i.e., PO7 and PO8) for each search condition. These cue-elicited ERPs were lateralized at different time points, prior to an N2pc, making it difficult to decide if the lateralized ERPs were of a sensory or an attentional origin. Thus, they were not further analyzed.

To investigate cue-relative effects on target processing instead, differences in the P1 and N1 local peak amplitudes of ERPs elicited by targets at cued locations and targets at uncued locations (in invalid conditions) under same cue-target hemisphere conditions, respectively, were analyzed at target-contralateral and target-ipsilateral sites within the time range of the participants’ individual peaks (i.e., 8–130 ms and 130–200 ms, respectively). Here, only ERPs from the difficult color search condition with long ISI were used because target-elicited ERPs in all other experimental conditions were obscured by the cue-elicited ERPs.

**Fig. 3:**
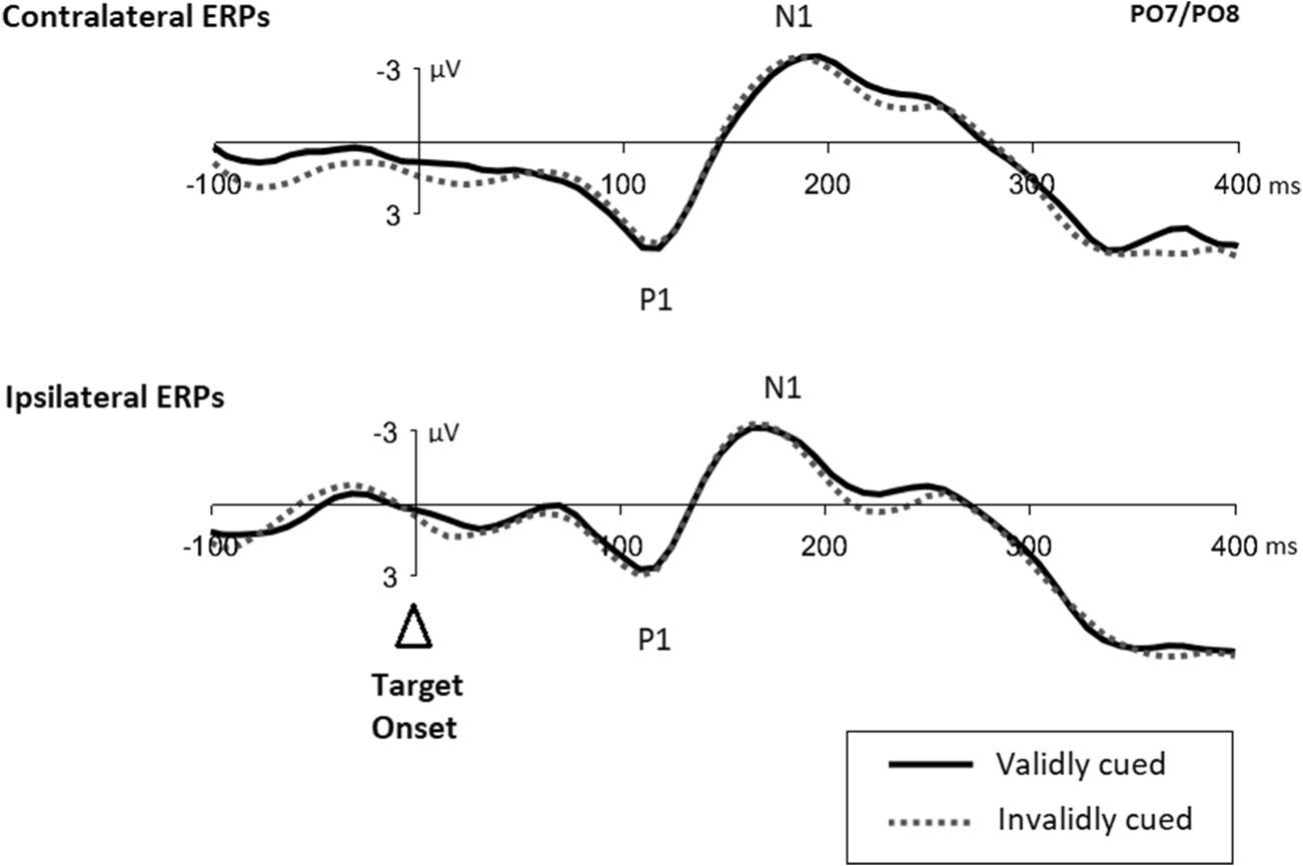
Event-related potentials for targets at validly cued and invalidly cued locations. *Note*. Target-elicited contralateral (top) and ipsilateral (bottom) event-related potentials (ERPs) in the difficult color search condition with ISIs of 200 ms. All graphs show the ERPs at posterior electrode sites PO7/PO8 with a baseline interval of 200 ms prior to cue onset. Negative values are plotted upward. Dotted lines show averaged ERPs from trials in which targets appeared at an invalidly cued location, solid lines from trials in which targets appeared at the validly cued position. Data were restricted to those invalid conditions in which the cue was presented on the same side as the target

Two repeated-measurements ANOVAs, with factors *hemisphere* (contralateral; ipsilateral) and *validity* (validly cued target location; invalidly cued location) were conducted on respective peak amplitudes. Figure 3 shows target-elicited contralateral and ipsilateral ERPs for validly cued and invalidly cued target locations.

##### ERP validity effects

The ANOVA on P1 amplitudes showed a significant main effect of *hemisphere, F*(1, 17) = 11.69, *p* = .003, η_p_^2^ = .41, with generally larger amplitudes observed over the contralateral (5.17 μV) than ipsilateral hemisphere (3.92 μV). There was neither a significant main effect of *validity, F*(1, 17) = 0.33, *p* = .573, η_p_^2^ = .02, nor an interaction between both factors, *F*(1, 17) = 2.78, *p* = .114, η_p_^2^ = .14.

The key question in regard to Gaspelin et al. (2016) was whether targets which appeared at the same location as a preceding cue generated an ERP validity effect. Therefore, we followed-up with two planned comparisons between levels of *validity* separately for different levels of *hemisphere* to specifically test for ERP cue validity effects. Lateral ERPs for validly cued locations showed no larger amplitudes compared with invalidly cued locations, neither at contralateral sites (validly cued: 5.5 μV; invalidly cued: 4.8 μV), *t*(17) = −0.86, *p* = .402, *d* = 0.21, *BF*_10_ = 0.37, nor at ipsilateral sites (validly cued: 4.0 μV; invalidly cued: 3.8 μV), *t*(17) = 0.20, *p* = .842, *d* = 0.05, *BF*_10_ = 0.25.

The ANOVA on N1 amplitudes showed no significant main effects of *hemisphere*, *F*(1, 17) = 0.09 , *p* = .768, η_p_^2^ = .01., or *validity, F*(1, 17) = 0.71, *p* = .410, η_p_^2^ = .04, nor an interaction between both factors, *F*(1, 17) = 0.45, *p* = .511, η_p_^2^ = .03. Two planned comparisons between levels of *validity* separately for different levels of *hemisphere* to specifically test for ERP cue validity effects were conducted. Again, lateral ERPs for validly cued locations showed no larger amplitudes compared with invalidly cued locations, neither at contralateral sites (validly cued: −4.9 μV; invalidly cued: −4.2 μV), *t*(17) = 1.25, *p* = .228, *d* = 0.13, *BF*_10_ = 0.48, nor at ipsilateral sites (validly cued: −4.6 μV; invalidly cued: −4.3 μV), *t*(17) = 0.48, *p* = .640, *d* = 0.08, *BF*_10_ = 0.27.

### Discussion

Neither behavioral, nor neurophysiological results from Experiment 1 showed clear evidence for attention capture by a salient, but task-irrelevant abrupt-onset cue. Importantly, we found no influence of task difficulty on behavioral capture effects, which is a central prediction of the attentional dwelling hypothesis (Gaspelin et al., 2016).

However, there are two caveats: First, search difficulty had a significant but small effect in Experiment 1, since mean RTs were only slowed by 19 ms to 26 ms on difficult search trials compared with easy search trials. For comparison, Gaspelin et al. (2016) found a difference of around 80 ms in mean RTs between difficult and easy search conditions in their Experiment 4. This might be one reason why we could neither detect a cueing effect under difficult search conditions in general, nor a significant difference in the size of cueing effects between search difficulties. Therefore, we assessed a sufficiently strong search difficulty manipulation prior to a follow-up EEG experiment (see Appendix A for the results of the behavioral pilot experiment). In addition, this time, we used RGB-color values in the target displays, exactly as reported in Gaspelin et al. (2016).^2^ Analyses regarding cue-relative ERP effects in the target display (e.g., ERP cue validity effects) thus become infeasible, but major hypotheses of the attentional dwelling explanation refer to the target-preceding time window between cue onset and target onset anyway. Furthermore, in Experiment 2, we left the target display on until participants responded via key press (cf. Gaspelin et al., 2016, Experiment 4), as we consider this discrepancy between Gaspelin et al.’s protocol and ours as potentially relevant for the observation of cueing effects in the difficult color search condition in Gaspelin et al. (cf. Kiss et al., 2012).

Second, in Experiment 1, the observed contralateral–ipsilateral differences in cue-elicited ERP components of potentially sensory origins make it impossible to distinguish between lateralized sensory- and attention-related ERPs (Luck, 2012; Luck & Hillyard, 1994). Therefore, we analyzed cue-relative effects of stimulus processing in the target-elicited ERPs only. Admittedly, this is not direct evidence for covert shifts of attention to the cues and prior to the targets. However, there was not much evidence of lateralized ERP effects or of any main effects of validity on target-elicited ERPs anyway.

## Experiment 2

Lateralized differences in cue-elicited ERPs of Experiment 1 have presumably originated from imbalances in stimulus energy between visual hemifields in the cueing display (e.g., Eimer, 1994; Luck, 2012; Luck & Hillyard, 1994; Mangun, 1995). This imbalance, however, cannot be avoided if we want to keep the design of the original search protocol of Gaspelin et al. (2016). Therefore, to provide some information on cue-elicited *direct* ERP modulations associated with attentional shifts toward the cue, in Experiment 2, we included another difficult color search block with abruptly onsetting, but task-relevant cues (i.e., matching the target color) to compare the ERPs of task-relevant and task-irrelevant cues. The assumption is that at least relevant/top-down matching cues will trigger spatial shifts of attention and that any difference in lateralized ERPs between relevant and irrelevant cues would be equated for sensory differences and would reflect attentional differences. To this end, the luminance (or energy or contrast) of the task-relevant and the task-irrelevant abrupt-onset cues was kept exactly the same in Experiment 2.

This rationale and the corresponding analysis and interpretations were based on implications from a recently published study of McDonald et al. (2022), who investigated neural correlates of visually guided orienting of attention (VOA) toward a single, unilaterally presented target stimulus. In a series of five EEG experiments, McDonald et al. isolated an ERP component indicative of visual orienting activity (VOA) reflected in an ipsilateral positivity over the occipital scalp in the time range of the P1 and N1 peaks. This was achieved by subtracting ERPs from conditions with no task-induced visual orienting activity from ERPs of conditions that triggered visual orienting activity toward a target stimulus. In rough analogy to McDonald et al.’s (2022) logic, we assumed that task-relevant cues triggered visual orienting activity starting at P1 latency. Consequently, if we find a difference in the cue-elicited lateralized ERPs between relevant and irrelevant conditions, the two irrelevant conditions can be compared for this difference to one another, too. In this way, we are able to at least find evidence for more attentional capture by irrelevant cues under difficult search than under easy search conditions, if such a difference existed. As explained in the Introduction, this would be a pattern of results in line with discrepancies between studies showing capture by irrelevant onset cues under difficult search conditions by Gaspelin et al. (2016) but also others (e.g., Adams et al., 2022; Lamy et al., 2018), and findings showing no capture by irrelevant onset cues under easy search conditions such as that of Goller et al. (2020). However, regarding the attentional dwelling hypothesis, one would expect no contralateral–ipsilateral differences in the analogous waveforms formed by subtracting ERPs of task-irrelevant cues under easy search conditions from ERPs of task-irrelevant cues under difficult search conditions. This prediction is based on the assumed similar degrees of attention capture by irrelevant onset cues in easy and difficult search conditions.

### Materials and methods

#### Participants

Twenty-three undergraduate psychology students from the University of Vienna took part in exchange for course credits (18 females; *M*_age_ = 21.3 years, *SD*_age_ = 2.4 years, range: 19–28 years). Exclusion criteria for the analysis of ERPs were the same as in Experiment 1, which were not met by three datasets leaving neurophysiological data from 20 participants (16 females; *M*_age_ = 20.9 years, *SD*_age_ = 1.8 years, range: 19–26 years).

#### Experimental setup and task

**Fig. 4:**
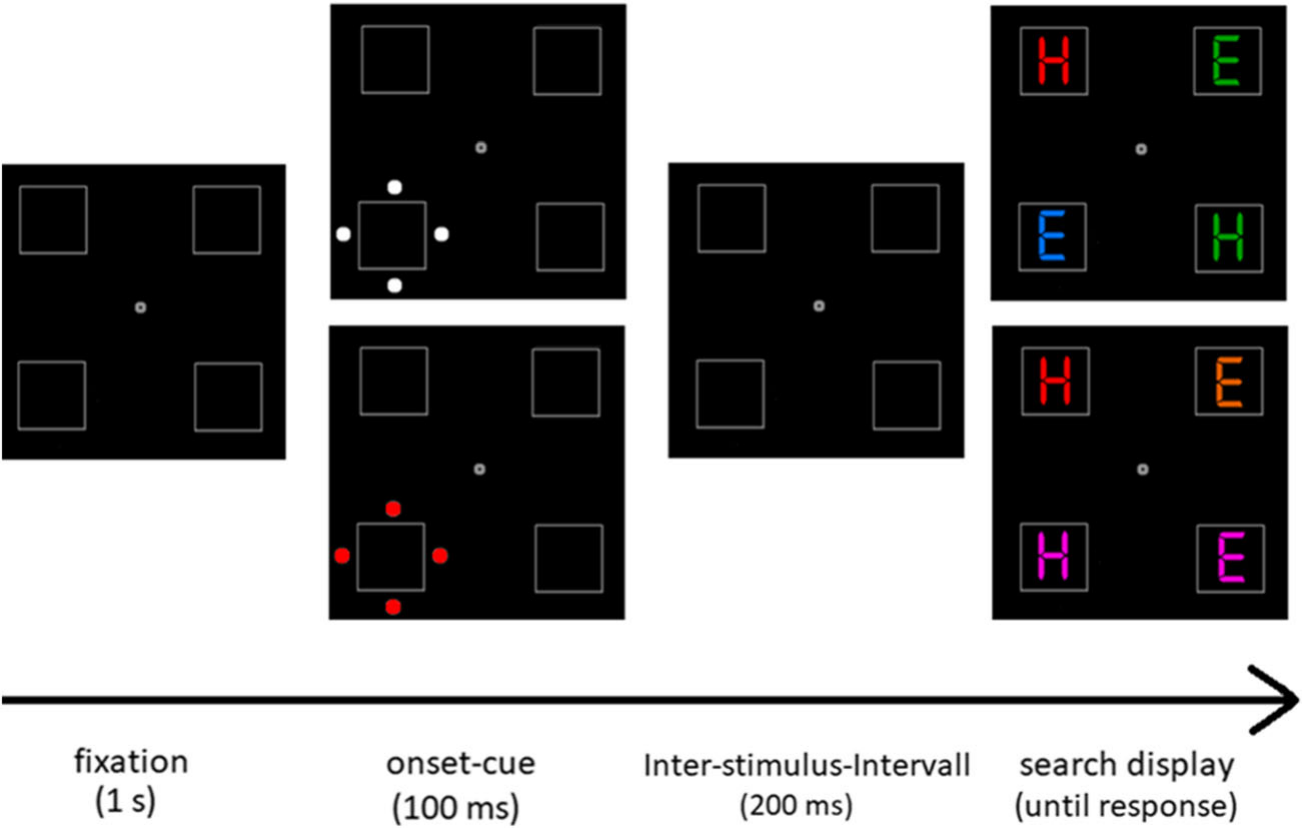
Experimental setup for Experiment 2. *Note.* Following a fixation screen, an abrupt-onset-cue was presented for 100 ms at one of the four placeholders. The cue was block wise either shown in “white” color and, therefore, task-irrelevant (Top, 2^nd^ from left), or in red color and, therefore, task-relevant (Bottom, 2^nd^ from left). (In fact, any luminance differences between cueing conditions were carefully avoided.) Following an interstimulus interval of 200 ms, a search display was presented until participants gave their response to red target letters via key press, *m* key for red *H*s and *y* key for red *E*s, respectively. Top (right): Exemplary target display in easy color search condition (i.e., distractor letters were blue and green). Bottom (right): Exemplary target display in the difficult color search condition (i.e., distractor letters were pink and orange). Note that stimuli are not drawn to scale and depicted in brighter colors than in the original experiment. (Color figure online)

The experimental setup and task were the same as in Experiment 1, except for four key changes (see Fig. 4). First, we used RGB color values for all stimuli in the target display as reported in Gaspelin et al. (2016), which are red (RGB 255, 0, 0), green (RGB 0, 151, 0), blue (RGB 0, 128, 255), pink (RGB 210, 0, 80), and orange (RGB 210, 80, 0). Second, we ran an additional difficult color search block, but with task-relevant cues (i.e., in red). Importantly, we carefully checked for equiluminant colors in all used cueing displays. Therefore, physical energies were kept constant between different search conditions to eliminate all sensory-related differences between ERPs from task-relevant and irrelevant cues (CIE L*a*b values for task-irrelevant cues: 55.4/−4.7/−25.4; task-relevant cues: 55.4/73.3/65.7). With this experimental design, any observable difference between relevant and irrelevant cueing conditions could be attributed to attention-related effects in the ERPs, and behavioral cueing effects could be taken to see which of the stimuli—relevant or irrelevant cues—captured more attention. Third, the search display remained on-screen until the participant gave a response via key press as in Gaspelin et al. (2016, Experiment 4).^3^ Fourth, we used again a slightly prolonged ISI of 200 ms to prevent any sensory interference from the onsetting target display in cue-elicited ERPs (see Fig. 4 for an exemplary trial sequence).

#### Electroencephalographic data recording and processing

Recording and processing steps on EEG data were the same as in Experiment 1. Among the final sets of 20 participants, 13.4% of all trials were removed by this procedure.

### Results

Behavioral data were preprocessed and analyzed as in Experiment 1.

#### Attentional capture (cueing effects)

##### Response times

The ANOVA showed significant main effects of *validity* (550 ms for valid cues; 564 ms for invalid cues)*, F*(1, 22) = 21.00 *p* < .001, η_p_^2^ = .49, and *search condition*, *F*(2, 44) = 19.60, *p* < .001, η_p_^2^ = .47, as well as a significant interaction between both factors, *F*(2, 44) = 6.00, *p* = .005, η_p_^2^ = .21. Participants responded generally faster in easy color search (525 ms) compared with difficult color search (580 ms with task-irrelevant cues; 579 ms with task-relevant cues). Post hoc *t* tests (α = .017; Bonferroni corrected for three comparisons) showed that differences between the easy search condition, and both difficult search conditions were significant, all *t*s > 4.48, all *p*s < .001, all *d*s > 0.90.

The key question in regard to Gaspelin et al. (2016) was the size of the cueing effect with task-irrelevant cues in general and in easy versus difficult search conditions with task-irrelevant cues in particular (see Table 3 for all mean RTs by *search condition* and *validity*). Here, task-relevant cues produced once more a substantially stronger numerical cueing effect compared with task-irrelevant cues. This was also reflected in the significant interaction in the ANOVA, and observed differences were subsequently confirmed by post hoc *t* tests between easy (irrelevant-cue) search and difficult (relevant-cue) search conditions, *t*(22) = 2.88, *p* = .009, *d* = 0.75. The observed difference in cueing effects between difficult search conditions, *t*(22) = 2.32, *p* = .030, *d* = 0.48, narrowly missed the level of significance after a Bonferroni correction for three comparisons (i.e., α = .017). Importantly, the considerably smaller numerical mean difference of 3 ms in cueing effects for task-irrelevant cues between easy and difficult search conditions was not significant, *t*(22) = 1.21, *p* = .238, *d* = 0.23, *BF*_10_ = 0.42.

**Table 3:** Mean response times (ms) by search condition and validity

	Easy color search with task-irrelevant cues	Difficult color search with task-irrelevant cues	Difficult color search with task-relevant cues
Invalid	527	583	584
Valid	518	571	562
Cueing effect	9	12	22

Cueing effects were calculated as invalid minus valid performance

##### Error rates

The ANOVA on arcsine-transformed error rates did neither show any significant main effects of *search condition* nor of *validity,* nor a significant interaction between factors, all *F*s < 1.24, all *p*s > .278, all η_p_^2^s < .05.

#### Attentional engagement (distractor compatibility effects)

##### Response times

Again, validly cued trials were not considered. The ANOVA showed only a significant main effect of *search condition*, *F*(2, 44) = 20.33, *p* < .001, η_p_^2^ = .48. Participants responded generally faster on easy color search trials (527 ms) than difficult color search trials (583 ms for task-irrelevant cues; 584 ms for task-relevant cues). Post hoc *t* tests (α = .017; Bonferroni corrected for three comparisons) showed these differences were significant, all *t*s > 4.8, all *p*s < .001, all *d*s > 0.91.

The key question in regard to Zivony and Lamy (2018) was whether distractors which appeared at the cued location in the target display generated a distractor compatibility effect. The ANOVA yielded neither a significant main effect of *distractor compatibility, F*(1, 22) = 0.35, *p* = .558, η_p_^2^ = .02, nor a significant interaction between *search condition* and *distractor compatibility*, *F*(2, 44) = 0.04, *p* = .931, η_p_^2^ < .01 (see Table 4 for distractor compatibility effects in all conditions). Already, here, we note the absence of a compatibility effect even with the task-relevant cues.

**Table 4:** Mean response times (ms) by search condition and distractor compatibility

	Easy color search with task-irrelevant cues	Difficult color search with task-irrelevant cues	Difficult color search with task-relevant cues
Incompatible	527	584	585
Compatible	526	583	583
Compatibility effect	1	1	2

Compatibility effects were calculated as incompatible minus compatible performance

##### Error rates

The ANOVA on arcsine-transformed error rates showed a significant main effect for *distractor compatibility, F*(1, 22) = 5.43, *p* =. 029, η_p_^2^ = .20, as well as a significant interaction between factors *distractor compatibility* and *search condition*, *F*(2, 44) = 5.50, *p* = .007, η_p_^2^ = .20. Post hoc *t* tests (α = .017; Bonferroni corrected for three comparisons) revealed a small but significant, reversed distractor compatibility effect for easy search trials with task-irrelevant cues (−1.9%), *t*(22) = 3.79, *p* < .001, *d* = 0.06.

#### Event-related potentials

**Fig. 5:**
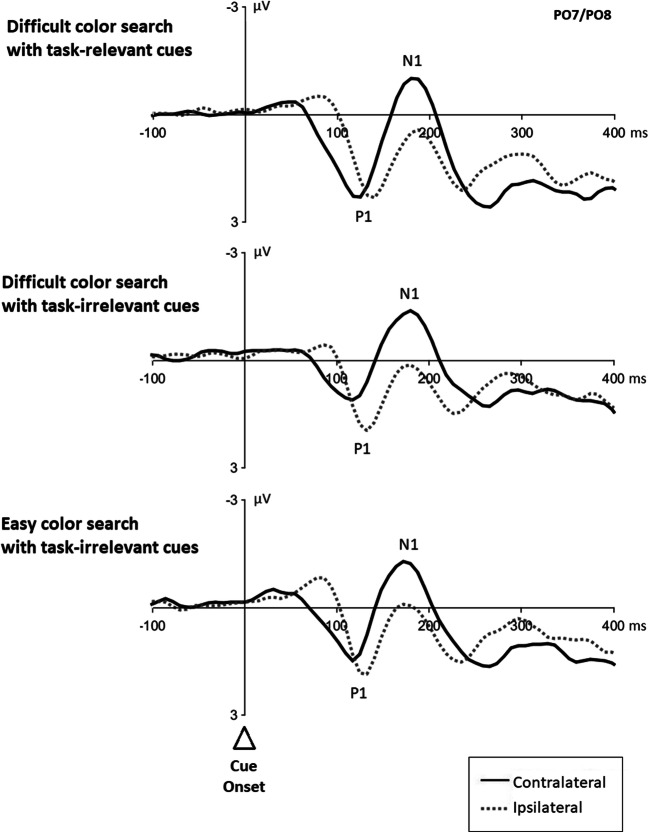
Cue-elicited event-related potentials from each search condition. *Note.* Cue-elicited event-related potentials (ERPs) for difficult color search with task-relevant cues (top), and task-irrelevant cues (middle), respectively, and easy color search with task-irrelevant cues (bottom). ERPs plotted time-locked to the onset of the cue. All graphs show the ERPs at posterior electrode sites PO7/PO8 (relative to a baseline starting −200 ms prior to the cue). Negative values are plotted upwards. The dotted lines illustrate the ipsilateral ERPs, the solid lines the contralateral ERPs

**Fig. 6:**
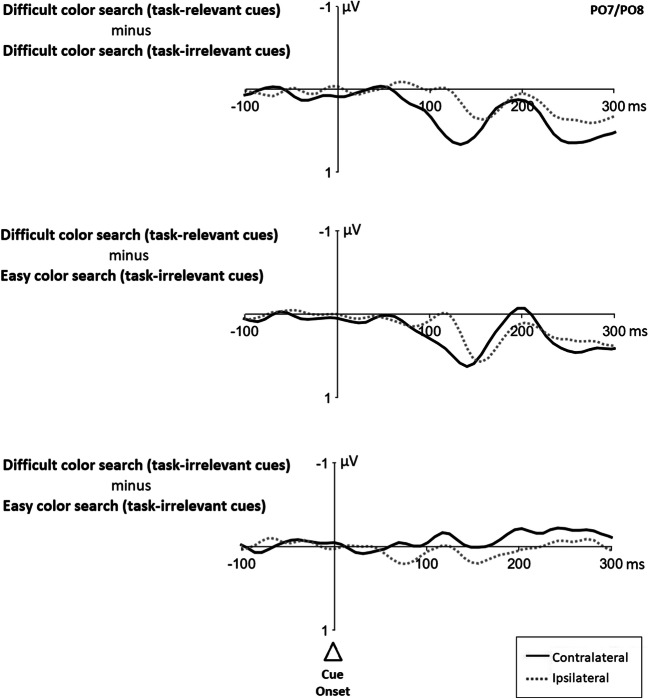
Difference waves between cue-elicited ERPs of all search conditions. *Notes*. Difference waves were computed by subtracting ERPs of task-irrelevant cues from ERPs of task-relevant cues (separately for contralateral and ipsilateral waveforms), upper two panels, and by subtracting ERPs of easy/task-irrelevant cues from ERPs of difficult/task-irrelevant cues (again separately for contra- and ipsilateral waveforms), lower panel. ERPs plotted time locked to the onset of the cue. All graphs show the ERPs at posterior electrode sites PO7/PO8. Negative values are plotted upwards. The dotted lines illustrate the ipsilateral ERPs, the dark lines the contralateral ERPs

Figure 5 shows cue-elicited ERPs at posterior contralateral and ipsilateral scalp sites (i.e., PO7 and PO8) for each search condition. As expected, we observed contralateral–ipsilateral differences starting at early latencies in the ERPs from both cue types, relevant and irrelevant cues. For the analysis on covert shifts of attention, we compared ERPs from task-relevant with task-irrelevant cues. Difference waves were computed by subtracting ERPs of task-irrelevant cues from ERPs of task-relevant cues (separately for contralateral and ipsilateral waveforms; see Fig. 6).

Following McDonald et al. (2022), mean amplitudes were measured in a 75 ms window spanning observed differences within the time range of contralateral and ipsilateral P1 and N1 peaks in each search condition with a respective baseline of 200 ms prior to cue onset (i.e., 80–155 ms). An ANOVA was then conducted on mean amplitudes with factors *hemisphere* (contralateral, ipsilateral) and *condition difference* (difficult color search with task-relevant cues minus difficult color search with task-irrelevant cues, difficult color search with task-relevant cues minus easy color search with task-irrelevant cues, difficult color search with task-irrelevant cues minus easy color search with task-irrelevant cues). Results showed a significant main effect of *hemisphere* (contralateral 0.28 μV, ipsilateral 0.14 μV), *F*(2, 38) = 9.19, *p* = .007, η_p_^2^ = .33, but no effect of *condition difference*, *F*(2, 38) = 4.02, *p* = .051, η_p_^2^ = .17, as well as an interaction between both factors, *F*(2, 38) = 17.36, *p* < .001, η_p_^2^ = .48. Post hoc *t* tests (α = .017; Bonferroni corrected for three comparisons) revealed significantly larger mean amplitudes at contralateral sites for differences between ERPs from task-relevant cues and task-irrelevant cues (easy search: contralateral 0.43 μV, ipsilateral 0.21 μV, *t*(19) = −3.00, *p* = .007, *d* = 0.53, *BF*_10_ = 7.03; difficult search: contralateral 0.48 μV, ipsilateral 0.10 μV, *t*(19) = −5.23, *p* < .001, *d* = 0.98, *BF*_10_ = 536.08). No contralateral–ipsilateral difference was observed for ERPs from difficult color search minus easy color search both with task-irrelevant cues, *t*(19) = 2.21, *p* = .040, *d* = -0.35, *BF*_10_ = 1.67.

### Discussion

The contingent-capture effect (Folk et al., 1992) for task-relevant colors is considered as very stable in reaction times and error rates, but it has been proven in the past that respective top-down matching onset cues also reliably led to ERP markers associated with the allocation of attention (e.g.,Goller et al., 2020 ; Lien et al., 2008). Therefore, we included another difficult color search condition but with task-relevant cues in our search protocol to investigate whether attentional processes contributed to early lateralizations in the cue-elicited ERPs, or whether cue-elicited lateralized ERPs merely reflected passive sensory registration of the cues. Analogous to McDonald et al.’s (2022) used methods in isolating the VOA toward a unilaterally presented target stimulus, we analyzed difference waves from conditions with task-relevant cues and task-irrelevant cues at lateral occipital sites. These ERP differences should be free of any sensory asymmetries as these were the same in all cueing conditions, so that the remaining lateralizations can be safely tracked down to attentional effects, especially where behavioral cueing effects would indicate a like difference between cueing conditions in their propensity to capture attention. Here, we found a contralateral positivity in the VOA time range in the relevant–irrelevant differences, irrespective of search difficulty with irrelevant cues. However, there was no corresponding difference between the two irrelevant cueing conditions. Thus, the current experiment shows a numerically stronger behavioral cueing effect with relevant than irrelevant cues that was accompanied by some cue-elicited attention-related lateralized ERP differences, but no differences between the smaller but significant behavioral cueing effects^4^ and the lateralized ERPs of irrelevant cues under easy versus difficult search conditions. While the latter ERP findings would be in line with the attentional dwelling hypothesis, the former similar behavioral cueing effects are at variance with this hypothesis. In addition, as in Experiment 1, there were also no distractor-compatibility effects,^5^ but this time they were also missing in relevant/top-down matching cueing conditions, indicating that response-compatibility effects are maybe not the most sensitive measure to test attentional engagement to begin with.

## General discussion

The present study used both behavioral and neurophysiological measures in a spatial-cueing protocol and showed that saliency per se is not sufficient for attentional capture by abrupt-onset cues during visual color search. Specifically, we did not find positive evidence for attention orienting activity toward task-irrelevant abrupt-onset cues prior to the search display in any of the ERP markers that we used. Neither did we find much influence of cue-target validity on target-elicited (lateralized) ERPs in Experiment 1, nor did we find positive evidence for direct stimulus-driven visual orienting activity in cue-elicited ERPs in Experiment 2. Results are, in this respect, in line with past neurophysiological evidence, concluding that only task-relevant abrupt-onset cues (i.e., that share target-defining properties) are capable of attentional capture (Goller et al., 2020; Lien et al., 2008). In the current study, this latter conclusion was also supported by positive evidence for cue-elicited visual orienting activity in task-relevant/top-down matching cueing conditions of Experiment 2.

### The challenge in studying unilateral abrupt-onsets in event-related potentials

However, the current study also demonstrates some of the intricacies of studying this question. A common criterion of attention allocation to cues is the presence or absence of a cue-elicited N2pc component indicated by a contralateral negativity over posterior scalp electrodes occurring roughly about 200–300 ms after stimulus onset (e.g., Luck, 2012; Luck & Hillyard, 1994). However, in the current study, we did not even get to analyzing this component. Instead, we observed earlier contralateral–ipsilateral ERP differences, which could likewise have originated from an imbalance in stimulus energy or sensory processing alone (Luck & Hillyard, 1994; Mangun, 1995; but see also McDonald et al., 2022; Yamaguchi et al., 1994). Hence, in Experiment 1, we abstained from analyzing any cue-elicited lateralized ERPs per se and analyzed only the target-elicited lateralized ERPs as a function of cue-target position relation (valid vs. invalid) in the long cue-target interval conditions. In this way, we wanted to see if attention capture by the irrelevant abrupt-onset cues had any consequences on target processing (e.g., Eimer, 1994; Hillyard & Anllo-Vento, 1998; Hopfinger & Mangun, 1998; Mangun, 1995; Mangun & Hillyard, 1991). We note the problems with this procedure, such as potential dependencies of just any target-elicited activity differences between cueing conditions not only on cue processing but also on target processing, meaning it would have been difficult to say if any of these ERP effects were elicited before target onsets. However, we found little indications for an improved perceptual processing of target stimuli at cued locations (under valid compared with invalid conditions) anyways, indicating that attention was probably not deployed to these cued positions.

To investigate *direct* ERP modulations elicited by the cues associated with attentional shifts, in Experiment 2, we included task-relevant cues in our search protocol that are known to reliably elicit covert shifts in attention (Goller et al., 2020; Lien et al., 2008). Only recently, McDonald et al. (2022) established a method for studying ERPs from abrupt onsets which allows one to isolate a component that separates activity of visually guided orienting in attention from sensory asymmetry effects. This visual orienting activity (VOA) is reflected in an ipsilateral positivity over the occipital scalp in the time range of the P1 and N1 peaks that McDonald et al. (2022) gained by subtracting the ERPs of unattended stimuli from the ERPs of attended stimuli. Analogously, we used expected contralateral–ipsilateral differences in the waveforms obtained by subtracting the ERPs of conditions with task-irrelevant abrupt-onset cues from the ERPs of task-relevant abrupt-onset cues, assuming that task-relevant abrupt-onset cues would capture attention at least more than task-irrelevant cues. In line with this assumption, we found a P1 positivity in the respective difference waves.

Although our assumption was, thus, generally confirmed, we found a contralateral instead of an ipsilateral positivity. To be precise, in line with McDonald et al. (2022), P1 was substantially larger for task-relevant cues (1.19 μV) than task-irrelevant cues (easy search 0.55 μV; difficult search 0.61 μV; *F*(2, 38) = 8.43, *p* = .001, ηp^2^ = .31; see Fig. 5). Importantly, however, at variance with McDonald et al., this was rather due to contralateral activity differences, as the ipsilateral P1 was not significantly larger for task-relevant cues (0.97 μV) than task-irrelevant cues (easy search 0.54 μV; difficult search 0.76 μV; all *t*s ≥2.10, all *p*s> .049, all *d*s < 0.60, cf. Fig. 5). Yet interpreting the presently found activity differences as attentional orienting differences was at least supported by also finding an (only numerically) larger cueing effect for the task-relevant cues than for the irrelevant abrupt-onset cues.

However, the procedures of our study did not allow studying of only one condition’s specific cue-elicited ERPs of the task-irrelevant cues alone (cf. McDonald et al., 2022). Thus, to test if an early attentional effect similar to that of the relevant cues existed in the irrelevant abrupt-onset cueing conditions, we could only subtract ERPs under easy/task-irrelevant conditions from ERPs under difficult/task-irrelevant conditions, providing a test bed for any stronger capture of irrelevant abrupt-onset cues under difficult than easy search conditions (see Appendix B for the results). However, there were no significant differences in lateral ERPs between these cue types (cf. Fig. 6). Thus, we found at least no positive evidence that a salient, but task-irrelevant abrupt-onset cue could have elicited covert shifts of attention. We note, however, that lacking differences in the lateralized ERPs elicited by irrelevant abrupt-onset cues would be in line with the attentional dwelling hypothesis.

### Alternative interpretations of the behavioral cueing effects

Importantly, we found no positive evidence for the attentional dwelling hypothesis in the behavioral cueing effects of Experiments 1 and 2. The attentional dwelling hypothesis predicts a stronger cueing effect of the irrelevant abrupt-onset cues under difficult than under easy search conditions. However, in the present Experiment 1, the irrelevant abrupt-onset cue created no behavioral cueing effects at all, and in the present Experiment 2, the cueing effects of the irrelevant abrupt-onset cues under easy and difficult search conditions were the same (for further evidence, see also the pilot experiment in the Appendix A).

The similarity of behavioral cueing effects under easy and difficult search conditions is not only at variance with the attentional dwelling hypothesis but also with other explanations of this difficulty-dependent cueing-effect difference such as the priority accumulation framework (PAF) model. According to the PAF, attentional priority accumulates over time and, thus, the use of the cue-location information awaits a trigger that signals the appropriate moment to use it (Darnell & Lamy, 2021; Lamy et al., 2018). Thus, the location of a task-irrelevant abrupt-onset cue might spatially bias processing priority, but, importantly, does not elicit a use of the corresponding cue information until task difficulty in the target display suggests considering these spatial priorities. According to PAF, mean RTs in a difficult search protocol might yield a cueing effect that is not reflective of an actual shift of attention toward the cued location and prior to the targets. Instead, the size of cueing effects would merely indicate how much accumulated cue position information is used to resolve the competition between the target and a cued distractor, as a function of search difficulty. Such post-target effects of spatial cues are in line with general evidence supporting that spatial cues can elicit some of their effects also on post-perceptual decisions rather than on target perception itself (cf. M. L. Johnson et al., 2020; Kinchla et al., 1995; Shiu & Pashler, 1994). However, as explained, as it stands, the predictions of PAF are at variance with the found similarity of cueing effects of irrelevant abrupt-onset cues under easy and difficult search conditions. Lastly, we highlight that the current study has not tested alternative search dimensions, such as letter search or shape search, which Gaspelin et al. (2016) showed produce even more powerful search difficulty effects. Thus, though we observed a stronger difficulty effect in Experiment 2 (and its pilot experiment in Appendix A) compared with Experiment 1, we acknowledge that it is peculiarly challenging to make a sufficiently difficult color search. Regarding the past evidence mentioned earlier, the influence of search difficulty on the size of behavioral attention capture effects cannot be completely disregarded without further testing with alternative search dimensions.

### Temporal duration of target displays as a moderating factor

What might have caused the general cueing effect of irrelevant abrupt-onset cues that we observed and that was independent of search-task difficulty in the present Experiment 2? First, we note that Experiment 1 does not pose a major problem for existing theories and explanations. In Experiment 1, the competition between the target and a cued distractor in difficult color search might have been too weak. The lacking cueing effects of the irrelevant abrupt-onset cues in Experiment 1 are, thus, relatively easy to explain, without having to assume additional factors on top of the ones proposed in the attentional dwelling hypothesis or PAF.

However, we found no difficulty-dependent cueing effect difference when a sufficiently strong search difficulty manipulation was realized in Experiment 2, too (see also Appendix A for additional supportive evidence). An important second factor (besides the stronger difference between easy and difficult search conditions) that we manipulated in the present Experiment 2 relative to Experiment 1 was search-display duration. Gaspelin et al. (2016) stated that a pilot experiment with target displays of 100 ms duration produced low accuracy in the difficult search condition, and therefore the authors allowed the target displays to remain on until participants gave a response in their main experiment. Yet this would not only have extended overall target display presentation times to about half a second but would have also allowed for relatively different search strategies. Importantly, participants could have used a partially sequential search strategy (i.e., more likely in difficult color search) consisting of a mixture of overt eye movements and covert attention shifts (Hulleman & Olivers, 2017; Treisman & Gelade, 1980) to enable enhanced foveal color perception to discriminate the red target letter among distractor letters (Gordon & Abramov, 1977; M. A. Johnson, 1986; McKeefry et al., 2007). To note, Gaspelin et al. did not control for eye movements in their study. In contrast, in the present Experiment 2 (and in Experiment 1), we carefully checked for overt eye movements by HEOG and VEOG, and trials with overt eye movements were excluded from the analyses. We therefore think that, in past studies, more eye movements under difficult search conditions than under easy search conditions could have selectively boosted cueing effects under difficult search conditions. For example, if eye movements in the direction of the cues were generally more likely under difficult search conditions, additional time to program or even execute a second saccade in invalid conditions might have increased the validity or cueing effect of irrelevant cues in past studies’ difficult search conditions compared with their easy search conditions.

At the same time, increasing target-display duration in Experiment 2 (compared with Experiment 1) and, hence, decreasing the necessity to select the target against a (generally nonpredictive) cue in close temporal proximity, might have relaxed observers’ self-imposed constraints on processing additional information, allowing generally more time for processing of accessory stimuli such as irrelevant abrupt onset cues (cf. Kiss et al., 2012). Thus, for a different reason than increased chances of eye movements, increased target-display durations might have fostered salience-based attentional effects of abrupt-onset cues in general (i.e., in difficult *and* easy search conditions) in Experiment 2 compared with Experiment 1. One should keep in mind though that, given the lack of positive evidence from ERPs that attention capture might be responsible for the cueing or validity effects of the irrelevant cues, we are inclined to believe that the irrelevant abrupt-onset cues become effective only following target onsets, in retrospect, for instance, in a manner more like sketched in PAF (though not leading to differences between easy and difficult search conditions in the color dimension).

### Habituation and suppression as moderating factors^6^

Could salient, but task-irrelevant abrupt onsets ever elicit involuntary shifts of attention? We consider this is possible but would depend on further critical side conditions. In a study on capture effects in a color search task, for example, Folk and Remington (2015) found that abrupt-onset cues captured attention, irrespective of any specific top-down set for abrupt onsets and, thus, at odds with contingent-capture theory (Folk et al., 1992). Crucially, for this type of attention capture, irrelevant abrupt onset singletons must be relatively rare events. Otherwise learning effects may result in rapid habituation obscuring the initial involuntary capture (cf. Neo & Chua, 2006; Turatto & Pascucci, 2016).

Relatedly, one might also want to consider the possibility that attention capture is the norm, and that active suppression might have been exerted in studies failing to show evidence of attention capture (such as the present one). For example, a recently proposed theory has suggested that, under certain conditions, a physically salient stimulus can even be actively suppressed to fully prevent involuntary attentional capture (i.e., the signal suppression hypothesis; for a review, see Luck et al., 2021). What we know now is that involuntary oculomotor capture by abrupt onsets cannot be suppressed: It can be reduced but not eliminated (Adams et al., 2022). However, as of yet, it remains open if suppression of covert attentional capture is possible and accounts for null findings with irrelevant abrupt-onset cues, when controlling for eye movements, as in the present study (cf. Goller et al., 2020; Lien et al., 2008).

## Conclusion

The current study shows that salience of abrupt onsets per se does not lead to attention capture. Neurophysiological results can close an important gap in the literature that opened the gate for speculations about potential shifts in attention toward salient, but otherwise task-irrelevant abrupt-onset cues that are hidden in the interval between cue onset and target onset. Behavioral results mainly supported a PAF model and, thus, challenged the dominating interpretation of cueing effects as covert shifts of attention. Furthermore, we found that the size of behavioral capture effects was not dependent on the proximity in color space between the stimuli in the search display, as stated by the attentional dwelling hypothesis and by PAF when eye movements were prevented. Importantly, with a post-target processing explanation on cueing effects, search display presentation times could be crucial. This finding is relevant, as arguably there are many situations in which the urgency to apply a top-down set is not that high, so that there is time and room for sampling even irrelevant information from the environment and for resulting memory-based attentional guidance by previously encountered irrelevant stimuli, too.

## Supplementary information

ESM 1 (DOCX 105 kb)

## Footnotes

1 Note that we had far more trials than were realized in the original study of `Gaspelin et al. (2016) to account for an EEG-compatible design. Thus, we analyzed both the first and the last 100 trials per block separately to enable comparisons in terms of cueing effects between the present RT data and the RT data reported by Gaspelin et al. We found that small quantitative but not significant cueing effects had developed only towards the end of the experiment, *F*(1, 22) = 2.62, *p* = .119, η_p_^2^ = .11, compared with the RT data for the first 100 trials, *F*(1, 22) = 0.41, *p* = .528, η_p_^2^ = .02. Hence, we rule out that cueing effects went undetected through more habituation (cf. `Turatto et al., 2018) in the current experiment than in Gaspelin et al.

2 Note that this led to physical differences in luminance between stimuli in the target displays, which is one reason why we had not already used them in Experiment 1.

3 We limited the time of target display presentation in Experiment 1 to 100 ms as in the other experiments in `Gaspelin et al. (2016), because we had achieved sufficiently good hit rates under difficult color search conditions in a small pilot prior to the main experiment. On the one hand, this might be attributable to the stimuli colors we used in Experiment 1, which were different to the ones used in the original experiment and, thus, resulting in a lower task difficulty. On the other hand, participants commonly show an overall better search performance in neurophysiological experiments than behavioral experiments. Thus, the experimental setting might also have led to improved hit rates.

4 The observed cueing effects of the irrelevant cues were somewhat smaller than in our pilot experiment (see Appendix A), which is most likely due to the prolonged ISI (Posner, 1980; Posner & Cohen, 1984).

5 We found a substantial distractor-compatibility effect for the relevant cues in our pilot experiment though (see Appendix A). We deem it likely that attentional priority weighting at the cued position faded over the time because of the prolonged ISI in Experiment 2, resulting in no longer observable response-compatibility effects in mean reaction times and error rates.

6 We thank our reviewers for raising these points to provide a more nuanced picture on attention capture by salient but otherwise task-irrelevant objects in visual search.
