# Supplementary material for: Novel tests of capture by irrelevant abrupt onsets: No evidence for a mediating role of search task difficulty during color search
Source: Atten Percept Psychophys. 2022 Dec 2;85(3):667–84. doi: 10.3758/s13414-022-02623-y (PMC9718589; doi:10.3758/s13414-022-02623-y)
Supplement: Supplementary file 1 — (DOCX 105 kb) [file 13414_2022_2623_MOESM1_ESM.docx]

**Appendix A**

**Assessment of Task Difficulty**

Prior to further EEG measurements, we ran a pilot experiment to assess behavioral attention capture effects (or lack thereof) with sufficiently high task difficulty.

**Materials and Methods**

***Participants***

Twenty-eight undergraduate Psychology students from the University of Vienna took part in exchange for course credits. Exclusion criteria for the analysis were the same as in Experiment 1. Thus, one dataset was excluded because the participant reported small deficits in color vision leaving data from 27 participants (19 females; *M_age_* = 20.6 years, *SD_age_* = 2.1 years, range = 18 to 26 years).

***Experimental Setup and Task***

Visual stimuli were presented on LCD monitors with a resolution of 1,280 x 1,024 pixels and a refresh rate of 100 Hz. A chin rest assured a viewing distance of 57 cm to the center of the monitor. The experiment was programmed and controlled via OpenSesame (Version 3.2.8; Mathôt et al., 2012) on PCs running Windows 7.

The experimental setup was the same as in Experiment 1, except for four key changes. First, we used RGB-color values exactly as reported in Gaspelin et al. (2016), which are red (RGB 255, 0, 0), green (RGB 0, 151, 0), blue (RGB 0, 128, 255), pink (RGB 210, 0, 80), and orange (RGB 210, 80, 0). Second, the search display remained on-screen until the participant gave a response via key press, exactly as in Gaspelin et al. (2016). Third, we ran an additional difficult color search block, but with task-relevant cues (i.e., in red). Fourth, ISIs were always 50 ms long (cf. Gaspelin et al., 2016, Experiment 4). Block order of the different cue-type conditions (easy search/task-irrelevant cue vs. difficult search/task-irrelevant cue vs. difficult search/task-relevant cue) was balanced across participants.

**Results**

Data were preprocessed and analysed as in Experiment 1.

***Attentional Capture (Cueing Effects)***

**Response Times**. The ANOVA showed significant main effects of *validity* (562 ms for valid cues; 589 ms for invalid cues)*, F*(1, 26) = 57.28 *p* < .001, η_p_² = .69, and *search condition*, *F*(2, 52) = 37.69, *p* < .001, η_p_² = .59. Participants responded generally faster in easy color search (533 ms) compared to difficult color search (610 ms with task-irrelevant cues; 607 ms with task-relevant cues). Post-hoc *t*-tests (α = .017; Bonferroni-corrected for three comparisons) showed that only observed differences between the easy search condition and both difficult search conditions were significant, all *t*s > −6.58, all *p*s < .001, all *d*s > 1.10.

The key question in regard to Gaspelin et al. (2016) was the size of the cueing effect with task-irrelevant cues in general and in easy versus difficult search conditions with task-irrelevant cues in particular. Observed cueing effects for task-irrelevant cues were of almost the same exact size in difficult and in easy search conditions (see Table 1A for all mean RTs by *search condition* and *validity*). This was the case, despite a substantial search-time difference between the difficult and easy search conditions. However, there was a substantially stronger cueing effect for task-relevant cues than task-irrelevant ones. This was also reflected in a significant interaction between factors *search condition* and *validity*, *F*(2, 52) = 50.50, *p* < .001, η_p_² = .66. Post-hoc *t*-tests (α = .017; Bonferroni-corrected for three comparisons) confirmed that the cueing effect was significantly stronger with task-relevant cues both than in easy search, *t*(26) = −7.89, *p* < .001, *d* = 1.92, and difficult search with task-irrelevant cues, *t*(26) = −7.65, *p* < .001, *d* = 1.69. Importantly, however, the difference in the size of observed cueing effects between search conditions with task-irrelevant cues was not significant, *t*(26) = −0.17, *p* = .866, *d* = 0.03, *BF*10 = 0.21.

**Table 1A**

*Mean Response Times (ms) by Search Condition and Validity*

Easy color search Difficult color search Difficult color search

(with task-irrelevant cues) (with task-irrelevant cues) (with task-relevant cues)

Invalid 536 613 565

Valid 524 601 622

Cueing effect 13 12 57

*Note.* Cueing effects were calculated as invalid minus valid performance.

**Error Rates**. Participants made numerically more errors in difficult color search (6.0% with task-irrelevant cues; 5.7% with task-relevant cues) compared to easy color search (4.4%), but differences between search conditions were not significant, *F*(2, 52) = 1.65, *p* = .202, η_p_² = .06. Furthermore, participants made significantly more errors on invalid trials (5.7%) than on valid trials (3.9%), *F*(1, 26) = 25.60, *p* < .001, η_p_² = .50. The interaction between factors *search condition* and *validity* turned out significant, *F*(2, 52) = 3.48, *p* = .038, η_p_² = .12. However, post-hoc *t*-tests (α = .017; Bonferroni-corrected for three comparisons) showed the same pattern as for the cueing effects in mean RTs. Significantly more errors were made on invalid trials with task-relevant cues both than in easy search, *t*(26) = −3.10, *p* < .005, *d* = 0.68, and difficult search with task-irrelevant cues, *t*(26) = −2.68, *p* < .013, *d* = 0.53. The difference between easy and difficult search conditions with task-irrelevant cues was again not significant, *t*(26) = −0.41, *p* = .688, *d* = 0.03.

***Attentional Engagement (Distractor Compatibility Effects)***

**Response Times**. The ANOVA showed a significant main effect of *search condition*, *F*(2, 52) = 43.55, *p* < .001, η_p_² = .63. Participants responded generally faster on easy color search trials (536 ms) than on difficult color search trials (613 ms for task-irrelevant cues; 622 ms for task-relevant cues). Post-hoc *t*-tests (α = .017; Bonferroni-corrected for three comparisons) showed that only the observed differences between easy search and both the difficult search conditions were significant, all *t*s > −6.4, all *p*s < .001, all *d*s > 1.08.

The key question regarding Zivony and Lamy (2018) was whether cued distractors in invalid conditions generated a distractor compatibility effect. Here, the ANOVA yielded no significant main effect of *distractor compatibility, F*(1, 26) = 1.59, *p* = .219, η_p_² = .06, but a significant interaction between *search condition* and *distractor compatibility*, *F*(2, 52) = 6.01, *p* = .004, η_p_² = .19. Post-hoc *t*-tests (α = .017; Bonferroni-corrected for three comparisons) showed a significant distractor compatibility effect only on difficult color search trials with task-relevant cues, *t*(26) = −3.2, *p* = .003, *d* = 0.19, *BF*10 = 11.38 (see Table 2A for all mean RTs by *search condition* and *distractor compatibility*).

**Table 2A**

*Mean Response Times (ms) by Search Condition and Distractor Compatibility*

Easy color search Difficult color search Difficult color search

(with task-irrelevant cues) (with task-irrelevant cues) (with task-relevant cues)

Incompatible 534 615 627

Compatible 539 611 613

Compatibility effect −4 3 14

*Note.* Compatibility effects were calculated as incompatible minus compatible performance.

**Error Rates**. The ANOVA on arcsine-transformed error rates did neither show a significant main effect of *search condition*, *F*(2, 52) = 2.44, *p* = .097, η_p_² = .09, nor of *distractor compatibility,* *F*(1, 26) = 0.83, *p* =. 372, η_p_² = .03, but an interaction between both factors, *F*(2, 52) = 22.05, *p* < .001, η_p_² = .46. Post-hoc *t*-tests (α = .017; Bonferroni-corrected for three comparisons) revealed a significant distractor compatibility effect (3.1%) on difficult search trials with task-relevant cues, *t*(26) = −4.1, *p* < .001, *d* = 0.17, as well as a significant, reversed distractor compatibility effect (−2.3%) on easy search trials with task-irrelevant cues, *t*(26) = 5.7, *p* < .001, *d* = 0.27.

**Discussion**

In this pilot experiment, we assessed a sufficiently strong search difficulty manipulation of 77 ms in mean RTs between easy and difficult color search conditions (with task-irrelevant cues; 74 ms with task-relevant cues) prior to a follow-up EEG experiment. For comparison, in Experiment 4 of Gaspelin et al. (2016) mean RTs between search conditions differed by about 80 ms. Other than in Experiment 1 of the current study, we observed significant cueing effects in all search conditions. As expected, task-relevant cues produced substantially larger cueing effects than task-irrelevant cues, which is support for top-down theories of attention capture contingent on human search goals (e.g., Folk et al., 1992). However, there was still no support for the Attention Dwelling Account in our pilot data. The Attentional Dwelling Hypothesis would have predicted a larger size of cueing effects from task-irrelevant cues under difficult than under easy search conditions. Although the present results do not provide evidence for the Attention Dwelling Hypothesis, they do support the Priority Accumulation Framework (PAF) Model (Lamy et al., 2018). According to PAF, information about cue locations is retrieved in target displays, once localizing the target is difficult. When human observers encounter difficulties during target search, they start exploiting biases of different origins, including cue positions, to direct their attention to alternative locations in a priority map that reflects the accumulated evidence acquired in the recent past for how likely it is that specific locations might contain a target. We found significant distractor compatibility effects only in trials with task-relevant cues indicating that attentional engagement is contingent on current search goals. This finding is entirely in line with Zivony and Lamy (2018), but not with Gaspelin et al. (2016).

**Appendix B**

**Follow-Up Analysis on Cue-Elicited Differences in Experiment 1**

For the sake of completeness, here are the results of follow-up tests on whether an early attentional effect like that of the relevant cues in Experiment 2 existed in the irrelevant, abrupt-onset cue conditions in Experiment 1.

***Event-Related Potentials***

Difference waves were formed between cue-elicited ERPs of all search conditions and separately for contralateral and ipsilateral waveforms (see Figure 1B). However, only differences between ERPs from search conditions with a cue-target time interval of the same length were subjected to a statistical analysis, that is, difficult and easy color search conditions with short ISI.

**Figure 1B**

*Difference Waves Between Cue-Elicited ERPs of all Search Conditions*

**
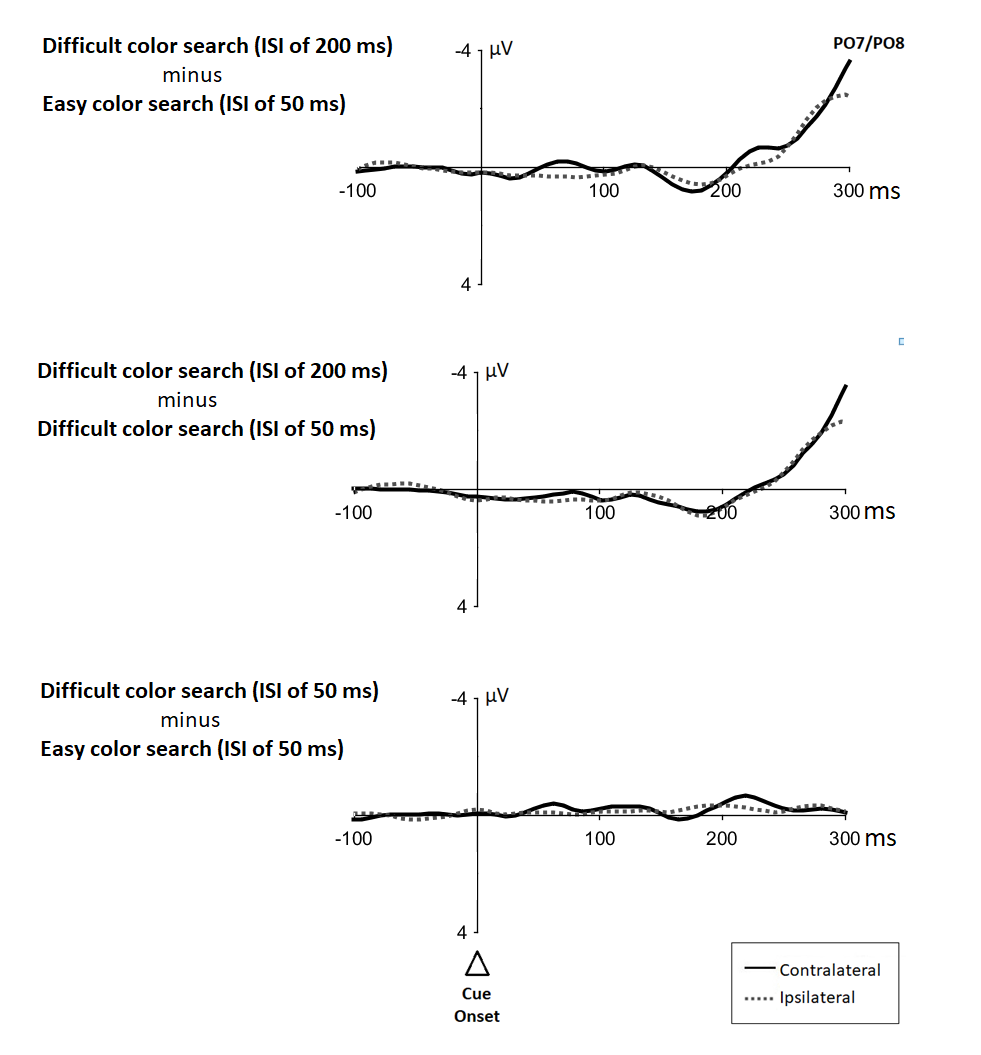
**

*Notes***.** Difference waves for cue-elicited event-related potentials (ERPs) from each search condition. ERPs plotted time-locked to the onset of the cue. All graphs show the ERPs at posterior electrode sites PO7/PO8. Negative values are plotted upwards. The solid lines illustrate the ipsilateral ERPs, the dotted lines the contralateral ERPs. The difference wave between search conditions with the same cue-target time interval, thus, relevant for the statistical analysis, is shown at the bottom.

Following McDonald et al. (2022), mean amplitudes were measured in a 75 ms window spanning observed differences within the time range of contralateral and ipsilateral P1 and N1 peaks in the respective search conditions (cf. Figure 2) with a baseline of 200 ms prior to cue onset (i.e., 75-150 ms). A *t*-test revealed no significant differences between contralateral and ipsilateral mean amplitudes in cue-elicited ERPs of difficult color search minus easy color search both with short ISI (contralateral −0.23 µV, ipsilateral −0.11 µV, *t*(17) = −0.74, *p* = .467, *d* = 0.14, *BF*10 = 0.31).
